# Pharmacological and Pharmacokinetic Insights Into Columbianadin: A Promising Natural Anti-Inflammatory Compound

**DOI:** 10.1155/mi/6284834

**Published:** 2025-09-29

**Authors:** Yusong Wang, Le Tang, Jia Miao, Shouxiang Cheng, Yanqing Xu, Wen Xie

**Affiliations:** ^1^Department of Pharmacy, The Second Affiliated Hospital of Wannan Medical College, Wuhu, Anhui, China; ^2^Pharmacology College, Anhui Xinhua University, Hefei, China

**Keywords:** anti-inflammatory, Columbianadin, pharmacokinetics, pharmacological effects, rheumatoid arthritis, traditional Chinese medicine

## Abstract

Columbianadin (CBN), a dihydroangelic acid ester and a naturally occurring coumarin compound, is primarily derived from plants in the *Apiaceae* family, notably the traditional Chinese medicinal herb *Angelica pubescens*. Modern pharmacological research has revealed that CBN exhibits a broad spectrum of bioactivities, including neuroprotective, antitumor, anti-inflammatory, antiarthritic, and analgesic effects. As a key bioactive constituent of *Angelica pubescens*, CBN demonstrates superior therapeutic potential compared to its source plant, attributed to its distinctive chemical structure and multitarget regulatory mechanisms. This review systematically summarizes the pharmacological properties and underlying molecular mechanisms of CBN, with a special emphasis on its emerging role in modulating inflammation and treating rheumatoid arthritis (RA) and related disorders. Additionally, the pharmacokinetic (PK) characteristics of CBN are discussed to support its potential development as a novel drug candidate. This work aims to provide a theoretical foundation for the rational design of natural product-based therapeutics and to promote the translational research and clinical application of CBN.

## 1. Introduction

*Angelica pubescens*, commonly known as “Duhuo” in traditional Chinese medicine (TCM), is the dried root of a plant belonging to the *Apiaceae* family. It has long been used for its effectiveness in dispelling wind and dampness, as well as relieving pain and stiffness associated with rheumatic diseases [1]. Historically, *Angelica pubescens* was first recorded in the *Shennong Bencaojing*. It has been valued as a highly effective anti-rheumatic and analgesic herb, with a documented history of use spanning nearly 2000 years in China for the treatment of joint pain, headaches, and related symptoms [2–4]. Today, it is widely cultivated in provinces such as Chongqing, Hubei, and Shaanxi. Modern phytochemical studies have identified various bioactive components in *Angelica pubescens*, including coumarins [5], volatile oils, and other constituents such as carotenoids, ferulic acid, and neochlorogenic acid [6, 7]. Among these, the coumarin compound dihydroangelic acid ester, also known as Columbianadin (CBN), has been recognized as one of the principal active constituents [8]. Notably, the 2020 edition of the *Chinese Pharmacopeia* lists CBN content as a quality control marker for *Angelica pubescens*, stipulating a minimum content of 0.080%, further underscoring its pharmacological significance. Compared to other coumarin derivatives such as Osthole, CBN exhibits stronger pharmacological activity, improved metabolic stability, and lower toxicity, making it a promising candidate for further drug development. In terms of pharmacodynamic (PD) properties, both CBN and Osthole retain the key lactone carbonyl group and aromatic ring system of the coumarin scaffold, providing a stable molecular conformation and ample sites for structural modification, thereby supporting the multi-target and multi-pathway pharmacological activities characteristic of coumarin derivatives. The unique α,β-unsaturated ester bond and oxygen-containing heterocyclic structure of CBN are likely key contributors to its distinct biological activity. The α,β-unsaturated ester bond enables covalent interactions with nucleophilic residues in biomolecules, while the dihydrofuran ring and oxygen bridge improve conformational flexibility and enhance coordination with protein targets [9–11]. These structural features, which are absent in Osthole, may account for CBN's stronger binding affinity and reactivity in biological systems. With the advancement of modern pharmacology, the multifaceted therapeutic potential of CBN has gradually come to light. Studies have demonstrated its anti-inflammatory, antitumor, antiviral, neuroprotective, and immunomodulatory effects [12, 13]. In particular, CBN shows greater therapeutic potential in the treatment of rheumatoid arthritis (RA) compared to other coumarin compounds [14].

However, current research on *Angelica pubescens* has largely focused on Osthole, and systematic evaluations of CBN remain limited [15]. This lack of comprehensive analysis has hindered a deeper understanding of the herb's component-specific mechanisms and pharmacological effects [16, 17]. Given CBN's broad biological activity and potential clinical relevance, it holds great promise for therapeutic development. This review therefore aims to systematically collect, organize, and summarize recent advances in the pharmacological properties of CBN, with a particular focus on its role in RA and related disorders. We hope this work provides a valuable reference for researchers and clinicians, and facilitates the further development and application of CBN in modern medicine.

## 2. Pharmacological Action and Mechanism of CBN

**Figure 1:**
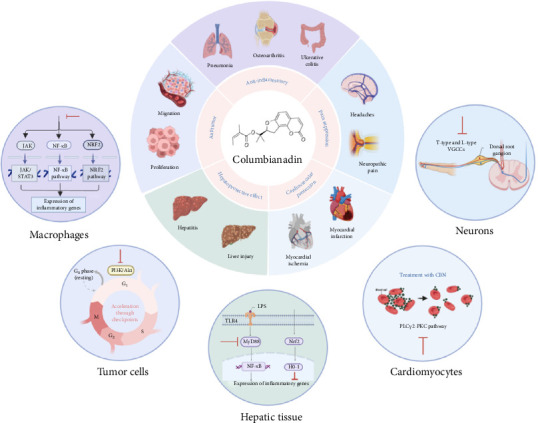
The main pharmacological activities of Columbianadin.

Modern pharmacological studies have demonstrated that *Angelica pubescens* exhibits significant antioxidant, anti-rheumatoid arthritis, and anti-Alzheimer's disease activities [18]. The traditional therapeutic effects of Chinese herbal medicine represent the culmination of long-term empirical knowledge accumulated by ancient practitioners and continue to inform modern clinical applications. For instance, the classical herbal formula Duhuo Jisheng Decoction is widely used in clinical settings for the treatment of RA [19–21] (Table 1). Pharmacokinetic (PK) studies of Duhuo Jisheng Decoction have shown that CBN and Osthole are among its principal constituents detected in the bloodstream [32, 33]. Beyond its clinical application in AR, the diverse pharmacological properties of CBN have been extensively validated in preclinical studies [34, 35]. In this study, we categorize these effects according to their pharmacological actions, as illustrated in Figure 1 and summarized in Table 2.

### 2.1. Anti-Inflammatory Effect and Mechanism

**Figure 2:**
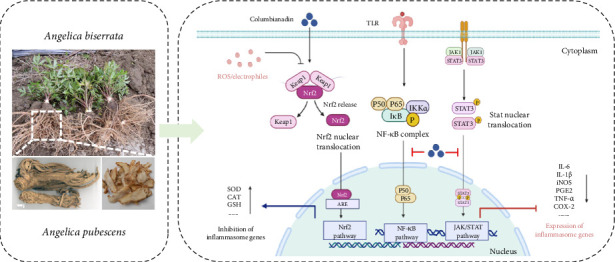
Anti-inflammatory mechanism of Columbianadin.

The anti-inflammatory mechanism of CBN primarily involves the regulation of key inflammatory signaling pathways and the suppression of pro-inflammatory cytokines such as TNF-α, IL-6, and IL-1β. By attenuating the production and release of these mediators, CBN effectively alleviates inflammatory responses, demonstrating therapeutic potential for various inflammatory diseases, particularly RA [4]. Experimental studies have shown that CBN significantly ameliorates arthritis symptoms in collagen-induced arthritis (CIA) mice, including reductions in paw swelling and arthritis scores, as well as modulation of inflammatory and oxidative stress markers [2, 36]. The anti-RA effects of CBN are primarily mediated through four mechanisms: inhibition of inflammation, regulation of oxidative stress, modulation of gut microbiota composition, and alteration of metabolic pathways. These effects are closely associated with the JAK1/STAT3, NF-κB, and Keap1/Nrf2 signaling pathways (Figure 2). Notably, Han et al. [37] demonstrated that CBN directly targets vimentin, thereby inhibiting activation of the VAV2/Rac1 signaling pathway. This action markedly reduces TNF-α-induced aberrant proliferation and migration of synovial fibroblasts, ultimately alleviating joint inflammation and mitigating pathological damage to cartilage and bone tissues in RA models. This newly identified mechanism expands the conventional understanding of anti-inflammatory agents, which typically focus on suppressing cytokine release. By targeting structural proteins and cell adhesion-migration pathways in synovial cells, CBN may offer a novel therapeutic strategy for RA.

In addition to its efficacy in RA models, CBN has demonstrated broad-spectrum anti-inflammatory potential across various inflammatory disease models, with its core mechanism largely attributed to the inhibition of the NF-κB signaling pathway. A series of in vitro and in vivo studies have confirmed that CBN suppresses NF-κB pathway activation and downregulates the expression of key pro-inflammatory cytokines [48, 58, 59]. In vitro experiments have revealed that CBN markedly attenuates lipopolysaccharide (LPS)-induced inflammatory responses and apoptosis in THP-1 cells by inhibiting the activation of the NOD1/NF-κB p65 signaling axis [38]. Further validation through NOD1 knockdown and overexpression experiments confirmed that the anti-inflammatory effects of CBN are indeed dependent on the regulation of this pathway. Mechanistically, CBN inhibits the phosphorylation and nuclear translocation of NF-κB p65, effectively reduces hydroxyl radical production in LPS-stimulated RAW264.7 macrophages, and modulates the expression of the antioxidant enzyme HO-1. In a DSS-induced ulcerative colitis rat model, CBN significantly reduced the expression of pro-inflammatory cytokines, including TNF-α, IL-1β, and IL-6, in colon tissues, while inhibiting activation of the TLR4/NF-κB signaling pathway. These effects collectively contributed to the attenuation of intestinal inflammation and oxidative stress injury [39]. In a D-galactose-induced liver injury model, CBN markedly downregulated the expression of TNF-α, IL-1β, and IL-6 in hepatic tissue and suppressed the activation of the JAK2/p38/ NF-κB axis, thereby alleviating liver inflammation and oxidative damage [40]. Similarly, in a rat model of acute reflux esophagitis, CBN reduced inflammatory cytokine levels by inhibiting the NF-κB pathway, effectively mitigating histopathological injury and modulating oxidative stress biomarkers such as malondialdehyde (MDA), glutathione (GSH), and superoxide dismutase (SOD) [41]. Moreover, in an airway inflammation model, CBN significantly suppressed the production of nitric oxide (NO) and IL-6 in LPS-stimulated alveolar macrophages and alleviated inflammation by downregulating iNOS expression, further reinforcing the role of NF-κB-dependent mechanisms in its anti-inflammatory activity [42, 60]. Notably, CBN not only suppresses acute immune activation by inhibiting classical NF-κB and MAPK signaling pathways but may also modulate states of chronic immune tolerance. For instance, Chen et al. [43] demonstrated that CBN significantly inhibits TNF-α-induced dendritic cell (DC) maturation, migration, phagocytosis, and T cell stimulatory capacity, thereby attenuating autoimmune inflammation while preserving immune homeostasis. This dual regulatory effect highlights the potential of CBN as a therapeutic candidate for immune-related disorders characterized by excessive inflammation or immune dysregulation, such as RA, inflammatory bowel disease (IBD), and graft-versus-host disease (GVHD), warranting further investigation.

Overall, the anti-inflammatory mechanism of CBN demonstrates typical characteristics of multitarget, multipathway, and multisystem regulation. Among these, NF-κB serves as the central signaling hub mediating its anti-inflammatory effect [61]. The regulatory effects of CBN on NF-κB p65 are primarily mediated through inhibition of upstream signaling pathways, such as the IKK/IκB axis or through disruption of key signaling complex formation. This leads to indirect suppression of p65 activation and nuclear translocation, thereby reducing the expression of downstream pro-inflammatory mediators regulated by NF-κB. Furthermore, the NF-κB pathway extensively interacts with other signaling cascades, including MAPK, STAT3, and NLRP3, which synergistically amplify inflammatory responses. Therefore, future studies should focus on mechanistic investigations into the role of CBN within the broader inflammatory signaling network, with particular attention to pathway hierarchy, target specificity, and cell type–dependent variations in response, rather than being confined to efficacy validation in specific disease models.

### 2.2. Antitumor Effect and Mechanism

In recent years, natural products and their derived small-molecule compounds have attracted widespread attention as promising sources of anticancer agents, owing to their unique chemical structures, low toxicity profiles, and multitarget regulatory capabilities. These properties have made them invaluable in both drug discovery and mechanistic studies for cancer therapy. Yang et al. [45] evaluated the antiproliferative activity of 11 coumarin-derived natural compounds in human bladder cancer E-J cells. Among them, CBN exhibited the most potent antitumor effect, displaying a clear dose–response relationship (IC_50_ = 2.30 × 10^−6^ mol/L). At a concentration of 10^−4^ mol/L, CBN completely inhibited E-J cell proliferation, while at 10^−5^ mol/L, it achieved an inhibition rate of 88.4%, indicating strong cytotoxicity and growth-suppressive effects. These findings suggest that CBN could serve as a promising lead compound for bladder cancer treatment. In brain tumor research, CBN has also demonstrated notable antitumor activity. Studies have shown that CBN induces G_0_/G_1_ phase cell cycle arrest and promotes apoptosis in glioblastoma (GBM) cells in a dose-dependent manner [46]. Mechanistic investigations revealed that CBN downregulates the PI3K/Akt signaling pathway and significantly inhibits tumor growth in an orthotopic GBM model. These results suggest that CBN not only regulates tumor cell proliferation and survival but may also possess a certain degree of blood–brain barrier permeability, highlighting its potential application in central nervous system malignancies. Similarly, in colorectal cancer models, CBN significantly inhibits the proliferation of HCT-116 cells [47]. Its antitumor mechanisms exhibit concentration-dependent effects on cell fate: at lower concentrations (25 µM), CBN primarily induces apoptosis, while at higher concentrations (50 µM), it tends to promote necrosis. Furthermore, CBN treatment markedly elevates intracellular reactive oxygen species (ROS) levels, disrupting redox homeostasis and further driving tumor cell death. These findings suggest that CBN may exert antitumor effects, at least in part, through oxidative stress modulation.

However, current research remains limited in scope. Most studies have utilized a small number of animal models, and investigations into CBN's roles in inducing tumor cell apoptosis, inhibiting proliferation, suppressing metastasis, promoting autophagy, and modulating the tumor microenvironment are still insufficient. There is a notable lack of advanced tumor models and comprehensive safety evaluations. For instance, the establishment of xenograft mouse models for efficacy validation and the use of flow cytometry to analyze immune cell dynamics within the tumor immune microenvironment have yet to be fully explored. On the other hand, the structural features of CBN provide a solid foundation for further optimization and drug development. The compound's characteristic furanocoumarin and angelic acid ester double-ring backbone presents a valuable scaffold for structural modification. Recent studies have demonstrated that substitution at the C-3′position of the benzene ring in CBN [47] can markedly enhance its selective cytotoxicity toward cancer cells while minimizing toxicity to normal cells. In future research, integrating molecular docking, quantitative structure–activity relationship (QSAR) modeling, and virtual screening approaches could facilitate the systematic identification of CBN derivatives with improved target affinity and pharmacological profiles. For example, structure-based optimization toward key targets such as TLR7 or PI3K may not only broaden the therapeutic indications of CBN but also improve its target specificity and clinical applicability. However, the antitumor mechanisms of CBN are not limited to its direct cytotoxic effects on tumor cells; they should also encompass its influence on the tumor microenvironment, modulation of immune responses, and other related pathways. For instance, Chen et al. [43] reported that CBN effectively inhibits TNF-α-induced DC maturation, migration, phagocytosis, and T cell-stimulating capacity. Whether low-dose CBN exerts immunosuppressive or even pro-tumor effects during antitumor immune responses remains to be further investigated.

### 2.3. Hepatoprotective Effect

The liver was the primary organ responsible for the metabolism of exogenous substances and played a critical role in regulating their biotransformation [1]. However, the metabolic conversion of xenobiotics into less toxic compounds could inadvertently lead to the generation of ROS, the formation of reactive metabolites, and the activation of signaling pathways involved in cell survival or death, all of which may contribute to liver injury [2]. As a result, identifying natural compounds with antioxidant, anti-inflammatory, and metabolic regulatory properties became a key focus in the field of hepatoprotection. In this context, CBN showed promising hepatoprotective effects in recent studies. Shi-hui et al. [49] employed a carbon tetrachloride (CCl_4_)-induced acute liver injury model in mice to evaluate the hepatoprotective effects of *Angelicae Pubescentis Radix* extract, which contained 1.46 mg/g of CBN. The hepatoprotective activity was primarily attributed to two mechanisms: first, activation of the Nrf2/HO-1 signaling pathway, which enhanced cellular antioxidant defenses by increasing GSH levels and SOD activity; and second, inhibition of the TLR4/MyD88/NF-κB pathway, leading to reduced levels of pro-inflammatory cytokines such as TNF-α, IL-6, and IL-1β in liver tissue, thereby alleviating inflammation and oxidative stress. However, as the extract also contained other bioactive constituents, including Osthole, the observed effects likely resulted from the synergistic action of multiple components. Thus, the specific contribution and potency of CBN within this context require further investigation. In follow-up studies, Jayakumar et al. [48] utilized an LPS-induced acute hepatitis mouse model to systematically assess the hepatoprotective activity of purified CBN. Their findings demonstrated that CBN significantly suppressed the expression of TNF-α, IL-1β, iNOS, and NO, reversed LPS-induced elevations in serum liver enzymes (AST and ALT), and markedly improved liver histopathology [48]. Collectively, these results suggest that CBN can effectively ameliorate acute liver injury caused by chemical toxins or bacterial endotxins through inhibition of classic inflammatory pathways and oxidative stress signaling.

In addition to its roles in modulating inflammatory and oxidative stress pathways, CBN also attracted growing interest for its potential regulatory effects on hepatic drug metabolism. Extracts from *Angelicae Pubescentis Radix* were reported to influence hepatic drug-metabolizing enzyme activity and alter the formation of metabolic intermediates, thereby indirectly contributing to hepatoprotection [62]. As a key quality control marker of *Angelica pubescens*, whether CBN exhibited similar regulatory activity on hepatic enzyme systems remains to be empirically confirmed. To date, no systematic studies had investigated the impact of CBN on the cytochrome P450 (CYP450) enzyme system, particularly on key metabolic enzymes such as CYP3A4. Given that many instances of drug-induced liver injury are driven by CYP-mediated metabolic activation and free radical production, CBN might exert hepatoprotective effects by modulating this enzyme system to reduce the generation of toxic metabolites or interrupt harmful metabolic processes. Further studies are warranted to elucidate the molecular mechanisms by which CBN may influence hepatic drug metabolism and its implications for liver safety and drug interactions.

### 2.4. Suppression of Neuropathic Pain and Arrhythmia

CBN exerts notable electrophysiological regulatory effects on both cardiomyocytes and peripheral sensory neurons by targeting multiple voltage-gated ion channels, particularly T-type and L-type voltage-gated calcium channels (VGCCs) and voltage-gated sodium channels. In the context of cardiac rhythm regulation, CBN has been shown to significantly inhibit Ca^2+^ influx mediated by L-type VGCCs in GH4C1 cells, establishing its pharmacological basis as a natural calcium channel blocker [63, 64]. Notably, its inhibitory effects on sodium channels in cardiomyocytes are concentration-, time-, and state-dependent. This suggests a potential “double-edged sword” effect on cardiac electrical stability, underscoring the need for precise dose control. Therefore, clarifying the relationships among its dose–response characteristics, bioavailability, tissue distribution, and cardiac electrophysiological parameters will be essential for advancing its development as a therapeutic agent.

In models of neuropathic pain, CBN effectively attenuates injury-induced neuronal hyperexcitability by significantly inhibiting T-type and L-type calcium currents. This reduction in calcium influx dampens nociceptive signal transmission and mitigates central sensitization, thereby alleviating hallmark symptoms of neuropathic pain, including spontaneous pain and mechanical hyperalgesia. Su et al. [51] employed an oxaliplatin-induced NP mouse model to systematically investigate the modulatory effects of CBN on pain behaviors and the electrophysiological properties of peripheral sensory neurons. Their findings demonstrated that CBN significantly alleviated both mechanical and cold hypersensitivity in treated mice. Electrophysiological recordings revealed that CBN markedly suppressed VGCC currents in dorsal root ganglion (DRG) neurons, particularly inhibiting T-type (low-threshold) and L-type (high-threshold) calcium channel activity. Notably, coadministration of the calcium channel blocker gabapentin antagonized the analgesic effects of CBN, further confirming that its mechanism of action is largely dependent on calcium channel inhibition. These results suggest that CBN may attenuate peripheral nociceptive input by modulating calcium channel-mediated electrical activity in DRG neurons. This electrophysiological mechanism provides a promising new avenue for the development of NP therapies based on the targeted regulation of ion channel function. Clinical experience and TCM research further support the analgesic properties of CBN. As the primary active compound in *Angelica pubescens*, CBN, together with coexisting coumarins, has been traditionally used to treat migraines, musculoskeletal pain, and rheumatic pain associated with wind-cold conditions. Experimental studies have demonstrated that ethanol extracts of *Angelica pubescens* can significantly prolong the latency period of acetic acid-induced writhing in mice, indicating a central analgesic effect [65, 66]. In the context of modern neuropathic pain research, pro-inflammatory cytokines and inflammatory kinases are known to play a critical role in neuronal sensitization and the maintenance of persistent pain. Li et al. [52] investigated the regulatory effects of coumarin-rich mixtures primarily composed of CBN and cnidic acid from *Angelica pubescens* on pain-related signaling pathways. Their results showed that these mixtures significantly inhibited mechanical hypersensitivity in a dose-dependent manner, reduced the expression levels of TRPV1 and phosphorylated ERK (pERK), and suppressed the release of inflammatory cytokines such as TNF-α, IL-1β, and IL-6.

In short, the analgesic effect of CBN in neuropathic pain is not limited to the alleviation of symptoms. It also reflects a comprehensive regulatory potential that involves multiple mechanisms, including the inhibition of electrophysiological ion channels, the modulation of inflammatory signaling pathways, and the suppression of sensory neuron sensitization. These effects highlight the value of CBN in advancing both the development and mechanistic understanding of natural analgesic compounds.

In the future, applying integrated research approaches such as patch-clamp coupled with omics analysis, multielectrode array techniques, and systematic screening of structural derivatives will provide a more comprehensive assessment of the therapeutic potential and pharmacological profile of CBN in complex pain models. This will provide important theoretical support and a molecular basis for the development of innovative analgesic drugs.

### 2.5. Cardiovascular Protective Effects

Modern medical research has revealed that the pathogenesis of myocardial ischemia is highly complex, involving multiple pathological processes such as impaired myocardial energy metabolism, dysregulation of cellular autophagy and apoptosis, the release of inflammatory mediators, and vascular endothelial dysfunction. Therefore, the identification of pharmacological agents capable of modulating these critical pathways holds significant value for the prevention and treatment of cardiovascular diseases. CBN has exhibited broad and effective cardioprotective effects across various cardiovascular-related experimental models. In studies on platelet function, CBN demonstrated potent anti-platelet activation properties. Specifically, it significantly inhibited human platelet aggregation induced by collagen stimulation, mainly by suppressing the PLCγ2-PKC signaling cascade and further downregulating the phosphorylation and activation of downstream effectors such as Akt and ERK/JNKs, ultimately attenuating platelet activation responses [54]. Moreover, recent evidence has shown that CBN can prolong the closure time of platelet thrombi under high shear flow conditions without affecting the bleeding time in normal mice. This finding suggests that CBN's antithrombotic effect exhibits both high selectivity and a favorable safety profile, highlighting its potential as a promising natural lead compound for the treatment of thromboembolic disorders [55]. In the context of myocardial ischemia-reperfusion injury, CBN has also demonstrated considerable therapeutic potential. It is worth noting that antithrombotic activity is often a critical mechanism in the prevention and treatment of complications associated with atherosclerosis. Previous studies have demonstrated that several coumarin compounds structurally similar to CBN, such as Osthole, exhibit pharmacological effects, including antiatherosclerotic activity and regulation of lipid metabolism [67, 68]. However, the role of CBN in these pathological processes has not yet been systematically investigated. Its potential in modulating lipid homeostasis and preventing atherosclerosis remains largely unexplored, highlighting significant value for future research and therapeutic development.

In a murine model of acute myocardial infarction established by Chang et al. [56] using mice, CBN treatment significantly reduced the infarct area. Mechanistic investigations revealed that CBN modulates the PI3K/Akt/mTOR signaling pathway and downregulates key autophagy-related proteins, including LC3, Beclin1, and Atg5. This modulation effectively inhibits excessive autophagy, preserves mitochondrial function, and maintains cardiomyocyte viability. The protective effects of CBN were notably diminished in the presence of the mTOR inhibitor rapamycin, confirming the central role of this pathway in its cardioprotective mechanism. In another study focusing on anthracycline-induced cardiotoxicity, CBN was shown to exert protective effects through the regulation of the Sirt1/FOXO1 signaling pathway. Peng et al. [57] demonstrated that CBN enhances the transcriptional activity of Sirt1 on FOXO1, leading to a significant reduction in ROS levels, attenuation of oxidative stress, and inhibition of cardiomyocyte apoptosis. The administration of the Sirt1 inhibitor Ex-527 reversed these protective effects, further supporting the involvement of the NAD^+^-dependent deacetylase Sirt1 in mediating CBN's actions. This pathway is particularly important for maintaining mitochondrial homeostasis and ensuring cell survival under oxidative stress, suggesting that CBN holds potential as a candidate molecule for interventions targeting myocardial oxidative injury.

In summary, CBN exerts multidimensional and multi-target pharmacological effects in cardiovascular protection, modulating key pathological processes including platelet activation, autophagy regulation, oxidative stress, and mitochondrial homeostasis. Although current studies on its roles in atherosclerosis and lipid metabolism remain limited, its structural characteristics and preliminary pharmacological data provide a strong foundation for further exploration. Future research should prioritize the development and application of human-relevant models to better replicate human pathophysiological conditions. For instance, the construction of cardiac or vascular organoids, as well as inflammatory coculture systems derived from human-induced pluripotent stem cells (iPSCs) or primary human cells, could be used to simulate CBN's pharmacological responses under myocardial ischemia, thrombosis, or immune-mediated inflammation. These systems would enable real-time assessment of CBN's effects on electrophysiological activity, metabolic function, and inflammatory signaling, thereby enhancing the translational significance of preclinical findings.

## 3. PK Study of CBN

Studying the PK and PD properties of CBN within the context of complex TCM formulations can provide valuable insights into the scientific basis of TCM. To date, limited PK studies of CBN have been conducted in rats, with the main parameters summarized in Table 3. The following sections will detail its absorption, distribution, metabolism, and excretion (ADME) characteristics.

### 3.1. Absorption

CBN exhibits rapid absorption and elimination in vivo (*T*_max_≈3.03h, *t*_1/2_≈5.15 h). As a fat-soluble compound, it possesses a certain degree of membrane permeability; however, its oral bioavailability remains relatively low. In healthy rats, the mean maximum plasma concentration of CBN following oral administration was 13.33 ± 25.37 ng/mL, with detectable plasma levels observed as early as 0.5 h postadministration [73]. Interestingly, when CBN was administered in the form of a crude extract, both its half-life and absorption rate were further reduced, likely due to other components in the extract promoting its ADME process. Wu et al. [75] conducted in vivo unidirectional intestinal perfusion experiments in rats and found that CBN could be effectively absorbed across all segments of the intestine, with the highest absorption efficiency observed in the colon. This finding indicates that CBN may be suitable for development as a slow-release or colon-targeted formulation. Moreover, across a concentration range of 19–186 μg/mL, CBN absorption did not exhibit concentration-dependent inhibition, and there were no significant changes in its absorption rate constant (Ka) or apparent permeability coefficient (Papp > 1 × 10^−6^ cm/s), suggesting a stable absorption profile. Yang et al. developed a reliable UPLC-MS/MS method to simultaneously assess the uptake of 16 coumarins both ex vivo and in vivo. Their results showed that all tested coumarins exhibited moderate to good uptake in Caco-2 cells (Papp > 1 × 10^−6^ cm/s), with CBN displaying the highest permeability (Pap*p*=26.22 × 10^−6^ cm/s). Notably, despite its relatively low content in the original extract, CBN reached the highest plasma concentration, which is presumed to result from the metabolic conversion of other precursor compounds [74].

### 3.2. Distribution

Current studies on the tissue distribution of CBN remain limited. However, available evidence indicates that following intravenous administration in rats, CBN is rapidly and extensively distributed to multiple organs, including the heart, lungs, kidneys, stomach, brain, and muscle. Its prodrug and primary metabolite, columbianetin (CBT), have also been detected in nearly all examined tissues [70, 76]. At 10 min postinjection, the order of tissue concentration was heart > kidney > lung > stomach > brain > muscle > spleen > liver, with the heart exhibiting the highest levels. This suggests that the heart may be a principal target tissue for CBN. The low concentration of CBN in the testes indicates minimal uptake in reproductive organs, accompanied by rapid clearance. In contrast, CBN levels in the intestine declined slowly, likely due to biliary excretion followed by accumulation in intestinal epithelial cells. Notably, CBN was also detected in the brain, peaking at 30 min postinjection, implying that it is capable of crossing the blood–brain barrier. Yang et al. [77, 78] further investigated the central distribution of various coumarins, including CBN, and demonstrated that CBN rapidly entered both cerebrospinal fluid (CSF) and brain tissue following administration. Its PK profile in the central nervous system displayed a bimodal pattern, similar to that observed in plasma, supporting the notion that CBN may exert pharmacological activity in the central nervous system, such as in the treatment of neuropathic pain or cerebral ischemia. In contrast, a study by Ge et al. [71] examined the tissue distribution of CBN following oral administration of 6 g/kg *Angelica pubescens* root (APR) extract. The distribution profile across most tissues exhibited a time-dependent increase followed by a gradual decline. In this study, the liver emerged as the primary site of CBN accumulation, with peak levels observed at 6 h postadministration [71].

### 3.3. Metabolism and Excretion

**Figure 3:**
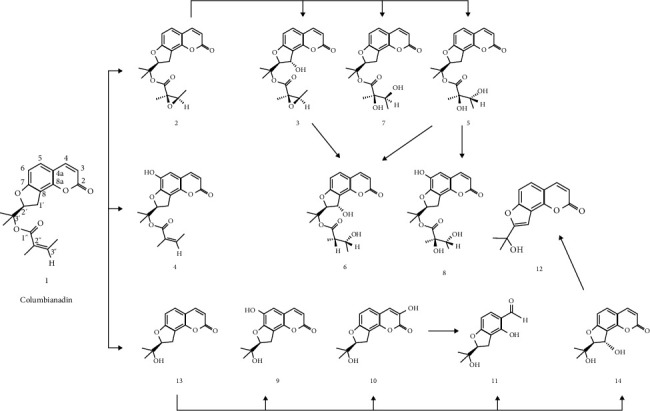
Major metabolites of Columbianadin.

Zhang et al. [58] identified 13 biotransformation products of CBN in vitro using a rat liver microsomal model (Figure 3). The primary metabolic pathways include hydroxylation, epoxidation, ester bond cleavage, and lactone ring opening. Most of these metabolites retained activity in inhibiting NO production, with IC_50_ values ranging from 42 to 73 μM. Among them, the parent compound CBN exhibited the strongest inhibitory effect, with an IC_50_ of 30.32 μM. Both CBN and its major metabolites significantly reduced NO production in LPS-stimulated RAW 264.7 mouse macrophage cells [58]. It is worth noting that PK studies of CBN under pathological conditions have not yet been reported. Disease states often lead to significant changes in physiological functions, including alterations in intestinal permeability, hemodynamics, plasma protein binding capacity, liver and kidney function, and enzyme system activity. These changes may markedly affect the PK of CBN in vivo, potentially altering both its efficacy and safety. Therefore, further research is needed to evaluate the PK behavior of CBN under disease conditions.

Overall, PK studies of CBN have preliminarily clarified its characteristics of rapid distribution and elimination, and suggest that hepatic and intestinal circulation may significantly influence its in vivo exposure. However, the interactions between CBN metabolites and other coexisting herbal components may affect its pharmacological efficacy or toxicity. The proportional composition and exposure profiles of CBN metabolites under multi-component synergistic conditions remain to be thoroughly investigated. The application of modern analytical technologies combined with systems biology approaches is expected to offer new insights into the material basis and mechanisms underlying its therapeutic effects. Moreover, the dynamic relationship between PK behavior and disease progression can be further explored through mathematical modeling. For example, Wang et al. applied a Duhuo-based gel patch to rabbits with an arthritis model and established PK parameters for Duhuo under pathological conditions. By using serum levels of IL-1β and TNF-α as PD indicators, they developed a PK-PD model to analyze the time-dependent changes in the major active components of Duhuo in RA model animals, thereby investigating the mechanism of action of acupoint-based delivery [79]. On this basis, future studies may incorporate physiologically-based PK (PBPK) models to integrate the physicochemical properties of CBN, its metabolic pathways, and interspecies physiological differences. This approach enables the rational extrapolation of animal data to humans. PBPK modeling can predict the PK behavior of CBN under pathological conditions, thereby improving the clinical relevance of preclinical findings, mitigating the limitations of traditional animal experiments, and supporting individualized therapy and clinical translation [80, 81].

## 4. Safety and Dosage Considerations

In vivo studies in mice have used CBN doses well below its reported toxicity threshold (LD_50_ > 2000 mg/kg) [36]. While short-term administration at specific concentrations has yielded promising results, the long-term safety and risks associated with high-dose use remain key challenges for clinical translation. As a typical coumarin derivative, CBN shares structural features with compounds known to cause mitochondrial dysfunction and elevated liver transaminase levels [82]. Moreover, CBN has been shown to inhibit cardiac sodium channels such as Nav1.5, raising concerns about potential cardiac side effects, including bradycardia and conduction block at higher concentrations [53]. On the other hand, the biological effects of CBN may display bidirectional regulatory properties across different cell types, with such variability influenced by multiple factors. First, different cell types may metabolize CBN into distinct active metabolites, resulting in substantial differences in biological outcomes. Second, CBN's effects may exhibit dose–dependent reversal, whereby low doses promote cytoprotection or exert antioxidant activity, whereas high doses induce oxidative stress and activate cell death pathways. Third, differences in experimental conditions are also critical; in vitro and in vivo studies differ markedly in metabolic milieu, drug exposure duration, and microenvironmental complexity, which may account for some of the discrepancies observed in reported findings. For instance, Kang et al. [47] reported that CBN induces a concentration-dependent increase in intracellular ROS, which may trigger distinct forms of cell death. Conversely, in models of inflammation or tissue injury, CBN acts as an antioxidant, mitigating ROS accumulation and protecting normal cells [48]. Similarly, tumor cells typically rely on aerobic glycolysis (the Warburg effect) and suppress mitochondrial ROS generation. CBN may counteract this suppression, thereby elevating oxidative stress. In contrast, cardiomyocytes depend heavily on mitochondrial respiration, and preserving mitochondrial homeostasis is essential for their survival. Under such conditions, CBN may mitigate stress-induced mitochondrial dysfunction, lower ROS levels, and ultimately exert a protective effect on myocardial cells. These findings underscore the importance of dose optimization and therapeutic window evaluation, which are essential for advancing the safe and effective clinical use of CBN.

## 5. Conclusions and Future Perspectives

TCM has accumulated extensive clinical experience over centuries, establishing a strong foundation of empirical safety and efficacy. Consequently, the exploration of natural bioactive compounds from TCM offers unique advantages for modern drug discovery and development [83–85]. CBN, a furanocoumarin analog isolated from *Angelica pubescens* and other TCM, has garnered increasing attention due to its multiple biological activities, including anti-inflammatory, antioxidant, neuroprotective, and immunomodulatory effects. Although existing studies have preliminarily shown that furanocoumarins participate in the regulation of classical signaling pathways such as MAPK, NF-κB, and JAK/STAT, there is still a lack of systematic understanding regarding their direct molecular targets, subcellular localization, and the integrative mechanisms connecting upstream and downstream regulatory pathways.

From a pharmacological perspective, the pleiotropic effects of CBN are likely not the result of broad, nonspecific actions but rather stem from a form of “network-based regulation” that involves a set of key molecular targets or signaling hubs. This mode of action is characteristic of multitarget compounds. For instance, CBN has shown strong inhibitory effects on inflammatory mediators such as TNF-α, IL-6, and iNOS in various inflammation models, suggesting that it may modulate upstream inflammatory sensors like NLRP3 and TLR4 [86], or key adaptor proteins such as MyD88 and TRAF6. In neuroprotection studies, CBN has been observed to mitigate oxidative damage, preserve mitochondrial membrane potential, and suppress caspase-3 activation. These findings imply that CBN may exert protective effects by regulating the mitochondrial-apoptosis axis. However, these pharmacological insights require further validation through advanced methodologies. Techniques such as chemical biology probe capture, single-cell transcriptomics, spatial genomics, and metabolic flux analysis can provide more precise and systematic insights into the molecular and cellular mechanisms of CBN action [87]. Notably, the presence of hydroxyl and carboxyl functional groups in CBN's structure confers a degree of hydrophilicity and reactivity, making it a promising candidate for the development of chemical probes. Drawing upon recent successes in “target fishing” strategies using probes derived from TCM compounds [88, 89], future studies could explore the design of CBN-based chemical probes. These could be employed to identify true cellular binding targets through techniques such as DARTS (Drug Affinity Responsive Target Stability), CETSA (Cellular Thermal Shift Assay), and TPP (Thermal Proteome Profiling). Such approaches would help establish a direct causal relationship between the small molecule structure of CBN and its target functions, thereby addressing key gaps in mechanistic understanding. It must be acknowledged that the current research on the pharmacological effects of CBN lacks validation in advanced models, which limits its ability to accurately replicate the complexity of human diseases. For example, most currently used mouse models are based on acute induction and therefore fail to fully capture the progression, complexity, and systemic nature of chronic human diseases. Future studies should incorporate humanized mouse models, 3D organoids, or microphysiological systems to enable more in-depth and translational investigations into the therapeutic potential of CBN.

Studies on the PK of CBN remain relatively limited, with most investigations concentrating on its PD responses, such as anti-inflammatory and neuroprotective effects, rather than on a systematic analysis of its ADME properties. Based on its chemical structure, it is hypothesized that CBN may encounter several PK challenges following oral administration, including poor gastrointestinal stability, extensive first-pass metabolism, and high plasma protein binding. Existing data suggest that CBN exhibits a moderate half-life and low oral bioavailability in rats, which may partly explain its limited systemic persistence. To address these limitations, future studies should prioritize comprehensive profiling of CBN's metabolites, tissue distribution kinetics, and ability to cross the blood-brain barrier. Techniques such as ultra-high-performance liquid chromatography coupled with quadrupole time-of-flight mass spectrometry (UHPLC-QTOF-MS) offer high-resolution characterization of metabolites and metabolic pathways across various tissues and organs [90, 91]. Additionally, radiotracer-based approaches can be employed to monitor CBN's distribution and clearance in the central nervous system, providing crucial insights into its neuropharmacological potential. Pharmaceutical strategies should also be explored to enhance CBN's PK properties. These may include the development of advanced drug delivery systems such as nanomicelles, liposomes, solid dispersions, or prodrug derivatives to improve stability, solubility, and tissue-specific targeting [92]. For instance, formulating CBN into nanocarriers capable of crossing the blood-brain barrier could significantly improve its accumulation and duration of action in brain tissue, thereby enhancing its neuroprotective efficacy in models of neurodegenerative diseases.

In summary, CBN as a bioactive constituent derived from TCM, possesses a strong pharmacological foundation and considerable potential for drug development. However, current limitations such as unidentified molecular targets, unclear mechanisms of action, and incomplete PK characterization continue to hinder its translational application. Future studies should employ multidisciplinary approaches that combine methods from omics technologies, structural biology, and chemical biology to systematically investigate CBN's mechanisms of action and behavior in vivo. Special attention should be given to elucidating its PK characteristics and their relationship with pharmacological efficacy [93, 94]. These efforts will provide a solid basis for advancing the clinical translation of CBN in the treatment of inflammatory and neurodegenerative diseases.

## Figures and Tables

**Table 1:** Origin and natural sorce of columbianadin [22–24].

S. No	Biological source	Inflammation-related diseases
1	*Angelica biserrata*	Inflammation, arthritis, and headache [22, 25]
2	*Angelica dahurica*	Headache, toothache, and furunculosis [26, 27]
3	*Angelicae Pubescentis*	Arthritis with pain in the lower back and knees, and headache [3, 18]
4	*Notopterygii rhizoma*	Arthritis, scapulohumeral periarthritis, and migraine [28, 29]
5	*Peucedanum decursivum*	Headache, fever, and rheumatoid arthritis [30, 31]

**Table 2:** Basic information on experiments related to the pharmacological effects of Columbianadin and its mechanisms.

Refs	Research model	Phenotype/pathways	Columbianadin dose (way)	Effect
**Anti-inflammatory effect and mechanism**				

[36]	Collagen-induced arthritis (CIA) mice	Inhibiting inflammatory response, regulating oxidative stress; JAK1/STAT3, NF-κB and Keap1/Nrf2	Orally administered with 20 mg/kg CBN	TNF-α, IL-6, IL-10, IL-1b↓ GSH, SOD↑
[37]	Collagen-induced arthritis (CIA) mice Cell: MH7A cells	Inhibit vimentin expression and function VAV2/Rac-1↓	Orally administered with 2 mg/kg CBN Cell: 12.5, 25, 50 μM	Rac-1↓
[38]	LPS-induced inflammatory model in THP-1 cells	Inhibits the NOD1/NF-κB p65 axis to suppress pro-inflammatory signaling.	Treated with CBN at concentrations of 30, 50, and 100 µg/mL for 24 h	TNF-α, IL-1β, MCP-1↓
[39]	DSS-induced colitis model in rats	Via alteration of HO-1/Nrf2 and TLR4-NF-κB signaling pathway	Orally administered with 5, 10, 15 mg/kg CBN	COX-2, PGE2, iNOS, TGF-β, IL-1β, IL-6, TNF-α ↓
[40]	D-Gal-induced liver injury model in mice	Via the JAK2/STAT3 and JAK2/p38/NF-κB Pathways	Orally administered with 200, 400, 800 mg/kg CBN	TNF-, IL-1β, IL-6, ALT, AST↓ SOD, CAT, GSH ↑
[41]	Acute reflux esophagitis model in rats	Inhibits nuclear translocation and DNA binding activity of NF-κB	Orally administered with 20, 50, 100 mg/kg CBN Cell: 5–80 µM	TNF-α, IL-6, IL-1β, iNOS, PGE2, COX-2 ↓
[42]	LPS-induced acute lung injury model	Suppression of proinflammatory enzyme expression	Orally administered with 20, 60 mg/kg CBN Cell: 100 μM	IL-6, NO, iNOS↓
[43]	TNF-α-induced maturation of dendritic cells	Via the TLRs/MAPKs/NF-κB pathway	3, 6, 13, 25, 50 μM	p38, p53, JNK 1/2, NF-κB↓
[44]	CAG was induced in rats by combined administration of MNNG and sodium salicylate.	Via the c-Fos/c-Jun pathway	Orally administered with 0.63, 1.26, 2.52 g/kg PC Cell:	TNF-α, IL-6, IL-1β, N-cadherin, Vimentin ↓ E-cadherin, SOX2, PG I, PG II ↑

**Antitumor effect and mechanism**				

[45]	Human bladder cancer cells	Inhibit tumor cell proliferation	10^−7^, 10^−6^,10^−5^, 10^−4^ mol/L	In a dose-dependent manner
[46]	Glioblastoma (GBM)	By suppressing PI3K/Akt signaling pathway	i.p. injections of CBN (20 mg/kg/day) Cell: 10, 20, 30, 40, 50 μM	Cyclin D1, Bcl-2, MMP2, MMP9↓ Bax, Cleaved-caspase 3↑
[47]	Human colorectal cancer cells	Inhibits colorectal cancer cell proliferation via apoptosis, necroptosis, and oxidative stress	12.5, 25, 50, 100 µM	Bax, p53↑ Bcl-2, Bim, Bid↓

**Hepatoprotective effect**				

[48]	LPS-induced liver injury model in mice	Inhibits NF-κB and MAPK (ERK/JNK) signaling pathways and reduces oxidative stress.	Intraperitoneally treated with 10, 20 mg/kg CBN Cell:20, 40 μM	TNF-α, IL-1β, iNOS ↓
[49]	CCl_4_-induced acute liver injury model in mice.	Via the Nrf2/HO-1 and TLR4/MyD88/NF-κB signaling pathways	Ethanol extract of *Angelica pubescens:* 50, 150, 300 mg/kg	TNF-α, IL-6, IL-1β, ALT, AST ↓ SOD, GSH ↑
[50]	CCl_4_-induced hepatotoxicity in rats, LPS-stimulated THP-1 macrophages	Significantly inhibits TNF-α expression	10 mg/kg, i.p. Cell: 20 µM	TNF-α, ALT, AST ↓

**Suppression of neuropathic pain and arrhythmia**				

[51]	Oxaliplatin-induced neuropathic mice	Inhibits T-type and L-type voltage-gated calcium channels	1, 3, 10 mg/kg, i.p. Cell: 1, 3, 10, 30 μM	Mechanical allodynia and cold hyperalgesia ↓
[52]	Spared nerve injury (SNI) model of neuropathic pain	Inhibit inflammatory cytokine expression, upregulate TRPV1 expression, and suppress ERK phosphorylation.	Coumarins from Radix angelicae pubescentis: 5, 10, 20 mg/kg, intra-gastrically	TNF-α, IL-6, IL-1β ↓
[53]	GH_3_ and HL-1 cells	Inhibit sodium currents and other ion channels	3, 10, 30 μM	I_Na_, late I_Na_ ↓ Tefluthrin ↑

**Cardiovascular protective effects**				

[54]	ADP-induced acute pulmonary thromboembolism in mice, Human platelets	Via the Akt and ERKs/JNKs signaling pathways	Mice: 5, 10 mg/kg, i.p. Human platelets: 60, 80 μM	p-Akt, p-ERK1/2, p-JNK1/2, ATP ↓
[55]	ICR mice, human platelets	Inhibit the phosphorylation of NF-κB and MAPK.	Mice: 10, 20 mg/kg, i.p. Human platelets: 45, 90 μM	HO•, ERK1/2, JNK1/2, IκBα, p65 ↓ CT ↑
[56]	AMI was induced in mice by ligation of the LAD coronary artery	Via the PI3K/Akt/mTOR signaling pathway	Orally administered with 10, 20, 40 mg/kg CBN H9c2 Cell: 1, 10 μM	Beclin1, Atg5, LDH, ROS ↓ LVEF, p-PI3K/PI3K, p-Akt/Akt, p-mTOR/mTOR ↑
[57]	Doxorubicin-induced cardiotoxicity mouse model	Via Sirt1/FOXO1 signaling pathway	10 mg/kg/day, i.p.	LDH, MDA, ANP, BNP↓ LVEF, CAT, SOD, Bcl-2, Nrf2, HO-1↑

**Others**				

[34]	Ovariectomy (OVX) to model osteoporosis	By inhibiting S6K/FLNC/ITGβ3 pathway	Orally administered with 12.5, 25, 50 mg/kg CBN, Cell: 0.1, 1, 10 μM	TRAP, S6K, FLNC↓
[35]	Vero-E6 cells infected with SARS-CoV-2	Binding to the nucleotide-binding pockets (NBPs) of multiple SARS-CoV-2 proteins	10, 25, 50 μM	Percent inhibition ↑

Abbreviations: ADP, adenosine diphosphate; ALT, alanine aminotransferase; AMI, acute myocardial infarction; ANP, atrial natriuretic peptide; AST, aspartate aminotransferase; ATP, adenosine triphosphate; Bcl-2, B-cell lymphoma 2; BNP, brain natriuretic peptide; CAG, chronic atrophic gastritis; CAT, catalase; COX-2, cyclooxygenase-2; DSS, dextran sulfate sodium; GPx, glutathione peroxidase; HO-1, heme oxygenase-1; ICR, Institute of Cancer Research; IL-6, interleukin-6; iNOS, inducible nitric oxide synthetase; JNK1/2, c-Jun N-terminal kinase 1/2; LDH, lactate dehydrogenase; LEVF, left ventricular ejection fraction; PGE2, prostaglandin E2; LPS, lipopolysaccharide; MAPK, mitogen-activated protein kinase; MDA, malondialdehyde; MMP-2, matrix metalloproteinase-2; MNNG, N-methyl-N'-nitro-N-nitrosoguanidine; MPO, myeloperoxidase; mTOR, mechanistic target of rapamycin; NF-κB, nuclear factor kappa B; NOD1, nucleotide-binding oligomerization domain-containing protein 1; Nrf2, nuclear factor erythroid 2-related factor 2; PC, periostracum Cicadae; PI3K, phosphoinositide 3-Kinase; ROS, reactive oxygen species; SOD, superoxide dismutase; SOX2, SRY-box transcription factor 2; TGF-β, transforming growth factor; TLR4, toll-like receptor 4; TNF-α tumor necrosis factor-α.

**Table 3:** Columbianadin main pharmacokinetic parameters.

Year refs	Subjects	Dose	Columbianadin parameters			
			*T*_1/2_ (min)	*T*_max_ (min)	*C*_max_ (ng mL)	AUC_0−∞_ (ng/min/mL)
[69]	Rat (*n* = 5)	10 and 20 mg/kg, intravenous	20.1799 ± 1.6825	NA	NA	98.0611 ± 4.7838 μg min/mL
[70]	Rat (*n* = 10)	5 mg/kg, intravenous	0.52 h	NA	4653 μg/L	1344 μg/L × h
[71]	Rat (*n* = 6)	Angelicae pubescentis radix extract 6 g/kg, oral	NA	NA	NA	NA
[72]	Rabbit (*n* = 6)	20 mg/kg, oral	4.9 ± 0.4 h	0.5 ± 0.17 h	75.5±1.6 mg/L	39.6 ± 1.7 mg/L × h
[73]	Rat (*n* = 10)	25 mg/kg, oral	5.15 ± 4.58 h	3.03 ± 1.87 h	13.33 ± 25.37	47.95 ± 66.80 ng/L × h
[73]	Rat (*n* = 10)	Angelicae pubescentis radix extract oral	0.92 ± 0.36 h	0.55 ± 0.33 h	1.82 ± 0.64	3.23 ± 1.46 ng/L × h
[74]	Rat (*n* = 6)	Angelicae pubescentis radix extract oral	2.78 ± 0.60 h	4.40 ± 0.89 h	9.85 ± 2.95 μg/L	73.50 ± 18.95 μg/L × h

## Data Availability

Data sharing not applicable to this article as no datasets were generated or analyzed during the current study.
